# Supplementary material for: Conditional chemoconnectomics (cCCTomics) as a strategy for efficient and conditional targeting of chemical transmission
Source: eLife. 2024 Apr 30;12:RP91927. doi: 10.7554/eLife.91927 (PMC11060718; doi:10.7554/eLife.91927)
Supplement: Figure 3—source data 3. [file elife-91927-fig3-data3.zip › Figure 3 source data3 part 1.zip!/Figure 3-Source data 3-part1/1175-9-CRISPResso_Report_rtnd62ko.zip!/CRISPResso_on_1175_9/4c.Quantification_window_insertion_deletion_substitution_locations.pdf]

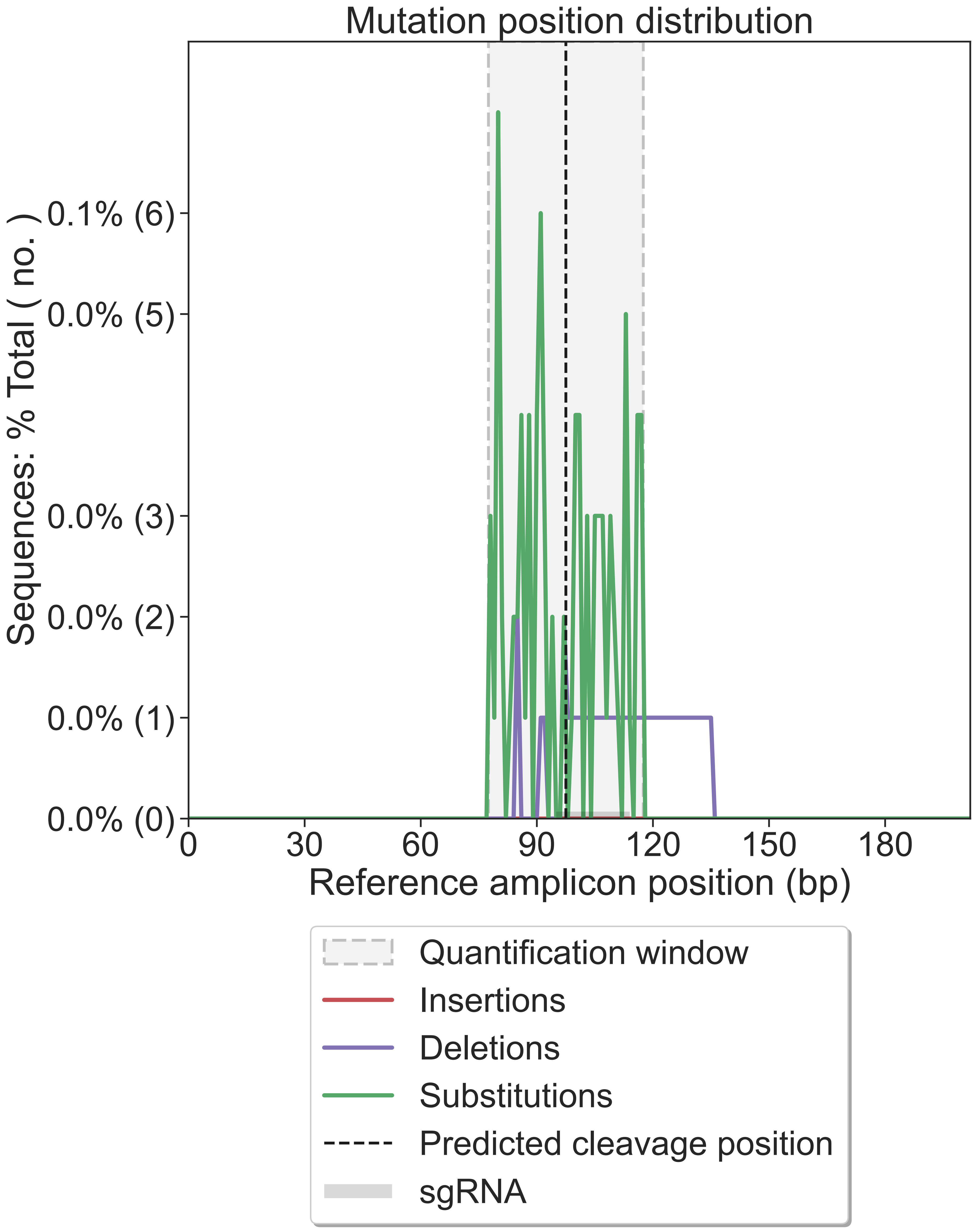
Mutation position distribution
Sequences: % Total (no.)
0.1% (6)
0.0% (5)
0.0% (3)
0.0% (2)
0.0% (1)
0.0% (0)
0
30
60
90
120
150
180
Reference amplicon position (bp)
Quantification window
Insertions
Deletions
Substitutions
Predicted cleavage position
sgRNA
